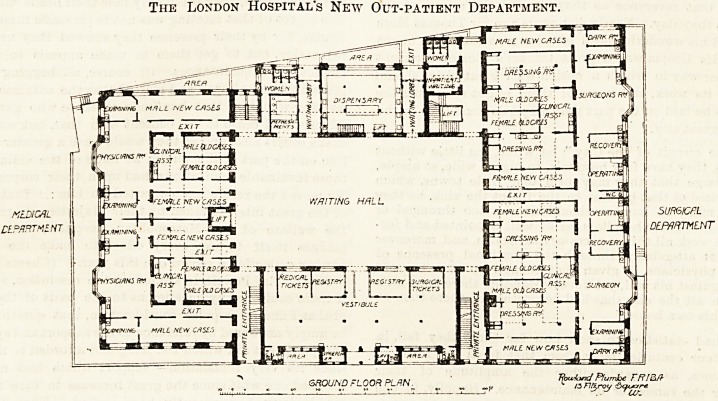# The New Out-Patient Department at the London Hospital

**Published:** 1903-06-13

**Authors:** 


### THE NEW OUT-PATIENT DEPARTMENT AT THE LONDON HOSPITAL.

The new out-patient buildings comprise a block to the west of the main part of the hospital, from which they are separated by Turner Street, under which there is a subway by which the two parts of the hospital are in communication. On going over the new building, one is struck at the first inspection by the admirable arrangements which have been made to avoid confusion in distributing the patients among the different departments—no simple matter where some six or seven hundred of them may be collected in a day. A patient coming in at the ordinary entrance, which is on the south side, finds himself in a vestibule, and here, if he has not attended before, his name is entered in the register, and he is given a book in which will be entered details concerning his case. When he has received this he passes on into the waiting hall, and thence to the department at which he is attending. Finally he passes out at the door opposite to the one at which he entered, leaving his book at the dispensary, where he receives at the same time any medicine that may have been ordered him. The waiting hall is a spacious and lofty room, with a glass roof supported on a steel framework. The floor is of mosaic, as are also those of the various corridors and waiting and consulting rooms. The walls of the waiting hall are lined for some eleven feet above the floor with glazed bricks, and above this are of unglazed red bricks. The room is said to be capable of seating upwards of 1,000 people, and there is a refreshment bar attached where patients may obtain light refreshments at a nominal price. Leading out of it are the surgeons' and physicians' rooms, with their accessory waiting and examining rooms. The east end of the ground floor is devoted to general surgical cases, and the west end to medical cases. The surgical department comprises two complete suites, each one consisting of a surgeon's and a clinical assistant's rooms, an operating room with a recovery room adjacent to it, two examining and separate waiting and surgical-dressing rooms. The rooms provided for the visiting staff are spacious, and provided with sufficient accommodation for a large number of students. The special departments, among which are the ophthalmic, aural, dental, orthopædic, obstetrical, and electrical, are upstairs on the first and second floors, and are approached by four staircases and two lifts. The stairs up to them are made of an artificial stone, which is roughened and prevents slipping. On each floor the separate departments are connected by corridors, which, as well as the rooms, are paved with mosaic, while the walls are for the most part lined with sirapite plaster. These special departments appear to be almost lavishly equipped with furniture and appliances of the most up-to-date and costly nature. A notable feature is the presence of a telephone in each portion of the building, which, through the hospital exchange, can afford communication with any part of the hospital or of the metropolis. By this means a great saving of labour is effected.

The building is fireproof, and heated throughout with radiators and hot-water pipes, there being no open fireplaces. Ventilation is ensured by shafts, through which the vitiated air is extracted by electric fans, and so far as a short visit was concerned there appeared to be neither stuffiness nor undue draught. The total cost of the building is £80,000, of which £25,000 was given by a friend of the hospital, while the remaining £55,000 still remains to be collected. The whole has been erected from the designs and under the superintendence of Mr. Rowland Plumbe, F.R.I.B.A., of 13 Fitzroy Square, London, W. Mr. Henry J. Wagg, 15 Great George Street, acted as Consulting Electrician, Mr. William Shepherd being the builder, and Mr. G. T. Murton, Clerk of Works. Anyone interested in the actual working of an up-to-date hospital could not do better than pay the London Hospital a visit.

THE LONDON HOSPITAL'S NEW OUT-PATIENT DEPARTMENT.

## Figures and Tables

**Figure f1:**